# Membrane Reactor for Methanol Synthesis Using Si-Rich LTA Zeolite Membrane

**DOI:** 10.3390/membranes11070505

**Published:** 2021-06-30

**Authors:** Masahiro Seshimo, Bo Liu, Hey Ryeon Lee, Katsunori Yogo, Yuichiro Yamaguchi, Nobuyuki Shigaki, Yasuhiro Mogi, Hidetoshi Kita, Shin-ichi Nakao

**Affiliations:** 1 Inorganic Membranes Research Center, Research Institute of Innovative Technology for the Earth (RITE), 1-7 Hikaridai, Seika-cho, Souraku-gun, Kyoto 619-0237, Japan; liu@njtech.edu.cn (B.L.); hr3638@naver.com (H.R.L.); yogo@rite.or.jp (K.Y.); yamaguti@osakagas.co.jp (Y.Y.); kita@yamaguchi-u.ac.jp (H.K.); nakao@rite.or.jp (S.-i.N.); 2 Steel Research Laboratry, JFE Steel Corporation, 1 Kokan-cho, Fukuyama 721-8510, Japan; n-shigaki@jfe-steel.co.jp (N.S.); y-mogi@jfe-steel.co.jp (Y.M.); 3 Environmental Science and Engineering, Graduate School of Sciences and Technology for Innovation, Yamaguchi University, 2-16-1 Tokiwadai, Ube 755-8611, Japan

**Keywords:** zeolite membrane, Si-rich LTA, methanol synthesis, membrane reactor

## Abstract

We successfully demonstrated the effect of a membrane reactor for methanol synthesis to improve one-pass CO_2_ conversion. An Si-rich LTA membrane for dehydration from a methanol synthesis reaction field was synthesized by the seed-assisted hydrothermal synthesis method. The H_2_O permselective performance of the membrane showed 1.5 × 10^−6^ mol m^−2^ s^−1^ Pa^−1^ as H_2_O permeance and around 2000 as selectivity of H_2_O/MeOH at 473 K. From the results of membrane reactor tests, the CO_2_ conversion of the membrane reactor was higher than that of the conventional packed-bed reactor under the all of experimental conditions. Especially, at 4 MPa of reaction pressure, the conversion using the membrane reactor was around 60%. In the case of using a packed-bed reactor, the conversion was 20% under the same conditions. In addition, the calculated and experimental conversion were in good agreement in both the case of the membrane reactor and packed-bed reactor.

## 1. Introduction

Methanol (MeOH) is an important chemical for acetic acid and formaldehyde synthesis. Generally, MeOH synthesis from syngas as H_2_, CO, and CO_2_ mixture is performed at high temperature (473–573 K) and high pressure (5–10 MPa) using Cu/ZnO as a catalyst in the industrial process. However, there is an issue that the one-pass yield of MeOH shows a low value owing to the limitation of equilibrium. A membrane reactor using MeOH and/or H_2_O permselective membrane is expected to improve one-pass yield. Struis et al. studied MeOH and H_2_O permeation performance from MeOH/H_2_ and H_2_O/H_2_ mixtures through Nafion membranes for a MeOH synthesis membrane reactor [1,2]. Nafion membranes show relatively high separation performance at 473 K. However, it is difficult to apply the membrane to a MeOH synthesis membrane reactor because of thermal stability and mechanical strength.

Inorganic membranes have thermal stability, chemical stability and mechanical strength. Pd-based [3,4], carbon [5,6], silica [7,8,9], and zeolite membranes are categorized as inorganic membranes. In particular, zeolite membranes have uniform micropores and selective adsorption, and their unique properties differ from that of other inorganic membranes. Zeolite membranes are often used for isomer separation owing to their uniform micropores [10,11,12,13]. Zeolite membranes can be used in the dehydration process, and several types of zeolite membranes such as LTA [14,15,16], MOR [17,18], and CHA [19,20,21] have been investigated for alcohol dehydration owing to their hydrophilic nature. In addition, LTA and CHA-types zeolite membranes with high performance for separation of alcohol/water mixture have been developed and commercialized. The LTA-type zeolite membranes show relatively high H_2_O flux compared with other types of zeolite membrane because theSi/Al ratio is 1. Okamoto and his co-workers reported that H_2_O flux through an LTA membrane was 3.5 kg m^−2^ h^−1^ at 378 K under water/methanol mixture conditions [14]. However, in generally, LTA membranes are difficult to apply methanol synthesis because of their hydrothermal stability. On the other hand, MOR [17] and CHA [21] zeolite membranes are expected to have high hydrothermal stability, however, H_2_O permeance is low to compared with LTA membranes. When inorganic membranes are applied to a membrane reactor for methanol synthesis, the membranes require high H_2_O and/or MeOH selective performance from H_2_O, MeOH, CO, CO_2_, and H_2_ mixture with high H_2_O and/or MeOH flux to improve the one-pass MeOH yield. In the case of MeOH removal from the reaction system, Na^+^-exchange ZSM-5 (Na-ZSM-5) membranes indicate permselective performance of polar molecules, such as H_2_O and MeOH, from the MeOH/H_2_O/H_2_ mixture in a relatively high temperature range [22]. Na-ZSM-5 membranes can be suitable for MeOH synthesis membrane reactors because Na cations, which are included in MFI-framework, have interaction with MeOH and H_2_O. However, a membrane reactor for MeOH synthesis from CO_2_ as a raw material requires a distillation process to obtain high purity MeOH, because both MeOH and H_2_O molecules can pass through that membrane. On the other hand, H_2_O permselective zeolite membranes that are applicable to the methanol synthesis membrane reactor have been developed and evaluated. Sawamura and his co-workers developed an MOR-type zeolite membrane and investigated the separation performance of the membrane under H_2_O-H_2_-MeOH mixture conditions [23]. H_2_O/MeOH permselective performance of the membrane showed a 73-101 of separation factor. Raso and his co-workers evaluated the H_2_O selective performance of several zeolite membranes such as LTA, T-type, CHA, and MOR under H_2_O/H_2_/CO_2_ mixture conditions [24]. According to this work, LTA zeolite membranes showed the best H_2_O/H_2_ separation performance for the methanol synthesis membrane reactor, and the H_2_O and MeOH permeances calculated from the exit gas composition of the membrane reactor were around 3.5 × 10^−8^ mol m^−2^ s^−1^ Pa^−1^ and 1.5 × 10^−9^ mol m^−2^ s^−1^ Pa^−1^, respectively.

To obtain high purity MeOH with a simple process, it is necessary to select a type of membrane reactor that extracts only H_2_O molecules, and the membrane requires high H_2_O permselective performance. Previously, we synthesized an Si-rich LTA-type zeolite membrane, which showed extremely high H_2_O/MeOH permselective performance [25]. In addition, the Si-rich LTA membrane had hydrothermal stability. In this study, we developed a membrane reactor for MeOH synthesis using an Si-rich LTA zeolite membrane to improve one-pass conversion compared with a conventional packed-bed reactor. A simulation model was also constructed to investigate optimal reaction conditions and membrane performance.

## 2. Materials and Methods

### 2.1. MeOH Synthesis Membrane Reactor Using an Si-Rich LTA Membrane

The Si-rich LTA zeolite membrane was synthesized by the seed-assisted hydrothermal synthesis method. The Si-rich LTA seeds prepared from a synthesis gels with 3.9Al_2_O_3_:18SiO_2_:5Na_2_O:173H_2_O, as suggested in the literature by Conato et al. [26]. Obtained seeds were coated on the symmetric alumina tube by rubbing method. The seeded support was soaked into the synthesis gels with 0.21Al_2_O_3_:SiO_2_:0.27Na_2_O:38H_2_O. Details of the synthesis conditions and methods are shown elsewhere [25]. The surface of the obtained membrane was observed by SEM, and the Si/Al ratio was measured by SEM-EDX (SU9000, Hitachi High-Tech Co., Tokyo, Japan). After membrane synthesis, vapor permeation performance of the membrane was evaluated under H_2_O/MeOH mixture conditions. The H_2_O/MeOH ratio was 10/90 wt% in vapor phase. The experimental apparatus was shown in our previous work [25]. H_2_O and MeOH flux were calculated using the following formula:

(1) $$J=\frac{m}{\left(A\times t\right)}$$

Where *J*, *m*, *A*, and *t* are flux (kg m^−2^ h), permeate weight (kg), the effective membrane area (m^2^), and the measurement time (h), respectively. The permeate vapor collected by the cold trap was injected into a GC-TCD (GC-4000 Plus, Shimadzu Co., Kyoto, Japan) for measurement of the H_2_O/MeOH molar ratio.

**Figure 1:**
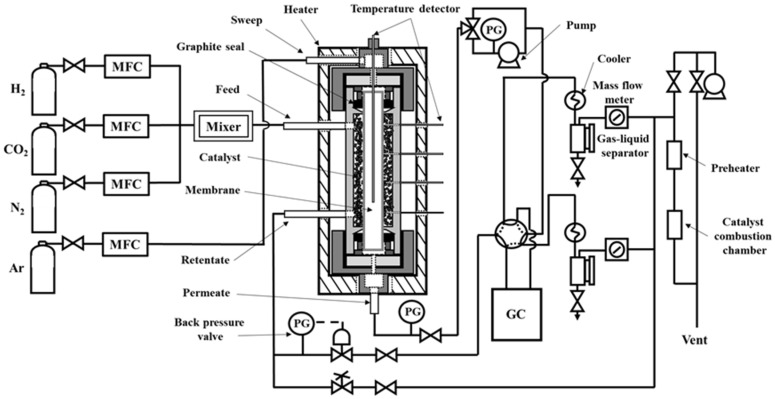
Schematic diagram of experimental apparatus for MeOH synthesis membrane reactor using an Si-rich LTA membrane.

The schematic diagram of the experimental apparatus for the MeOH synthesis membrane reactor is shown in Figure 1. Cu/ZnO catalyst was used for MeOH synthesis and placed outside of the membrane. The catalyst particle size was around 500 µm. The inner diameter of the reactor was 22 mm. The catalysts were conducted hydrogen reduction at 453 K under H_2_/N_2_ mixture condition overnight before the reaction tests. CO_2_ and H_2_ mixture gas, as raw material, was fed to the catalyst bed. The molar ratio of H_2_/CO_2_ was 3, which is the same as the stoichiometric ratio. The generated H_2_O was through the membrane, which was placed at the center of the reactor, and flowed to the permeation side. The CO_2_ conversion was calculated from the concentrations of CO_2_, H_2_, MeOH, and H_2_O in the retentate and permeate side. These concentrations were measured by gas chromatograph (GC-4000 Plus, Shimadzu Co., Kyoto, Japan). The membrane reactor tests were conducted under the following conditions: 453–493 K, 1–4 MPa, and SV = 200, 1000 h^−1^.

### 2.2. Simulation Model of Membrane Reactor for MeOH Synthesis

The MeOH synthesis from the hydrogenation of CO and CO_2_ mixed gas consists of the following CO_2_ conversion (1), CO conversion (2), and reverse water gas shift reaction (3).

(1) CO_2_ + 3H_2_ ⇄ CH_3_OH + H_2_O  ∆H = −49.4 kJ/mol
(2) CO + 2H_2_ ⇄ CH_3_OH       ∆H = −90.9 kJ/mol
(3) CO_2_ + H_2_ ⇄ CO + H_2_O      ∆H = 41.5 kJ/mol

In this study, we constructed a membrane reactor simulation using the kinetic model proposed by Graaf and co-workers for low-pressure methanol synthesis on a Cu/ZnO/Al_2_O_3_ catalyst [27,28,29]. The correspondent rate expressions due to the hydrogenation of CO_2_, CO, and the reverse water gas shift reaction are:

CO_2_ conversion

(2) $$r_{D}=\frac{k_{D}K_{{\mathrm{CO}}_{2}}\left(f_{{\mathrm{CO}}_{2}}f_{H_{2}}^{3/2}-f_{{\mathrm{CH}}_{3}\mathrm{OH}}f_{H_{2}O}/\left(f_{H_{2}}^{3/2}K_{D}^{0}\right)\right)}{\left(1+K_{\mathrm{CO}}f_{\mathrm{CO}}+K_{{\mathrm{CO}}_{2}}f_{{\mathrm{CO}}_{2}}\right)\left(f_{H_{2}}^{1/2}+\left(K_{H_{2}O}/K_{H_{2}}^{1/2}\right)f_{H_{2}O}\right)}$$

CO conversion

(3) $$r_{M}=\frac{k_{M}K_{\mathrm{CO}}\left(f_{\mathrm{CO}}f_{H_{2}}^{3/2}-f_{{\mathrm{CH}}_{3}\mathrm{OH}}/\left(f_{H_{2}}^{1/2}K_{M}^{0}\right)\right)}{\left(1+K_{\mathrm{CO}}f_{\mathrm{CO}}+K_{{\mathrm{CO}}_{2}}f_{{\mathrm{CO}}_{2}}\right)\left(f_{H_{2}}^{1/2}+\left(K_{H_{2}O}/K_{H_{2}}^{1/2}\right)f_{H_{2}O}\right)}$$

Reverse water gas shift reaction

(4) $$r_{R}=\frac{k_{R}K_{{\mathrm{CO}}_{2}}\left(f_{{\mathrm{CO}}_{2}}f_{H_{2}}-f_{H_{2}O}f_{\mathrm{CO}}/\left(K_{R}^{0}\right)\right)}{\left(1+K_{\mathrm{CO}}f_{\mathrm{CO}}+K_{{\mathrm{CO}}_{2}}f_{{\mathrm{CO}}_{2}}\right)\left(f_{H_{2}}^{1/2}+\left(K_{H_{2}O}/K_{H_{2}}^{1/2}\right)f_{H_{2}O}\right)}$$

where *r*_D_, *r*_M_, *r*_R_ represent the reaction rate of hydrogenation of CO_2_, CO, and reverse water gas shift, *k*_D_, *k*_M_, *k*_R_, the reaction rate constant, *K*_D_, *K*_M_, *K*_R_, the equilibrium constant, *K*_j_, the adsorption equilibrium constant of j component, and *f*_j_, fugacity. The equilibrium state between the reaction in the membrane reactor and H_2_O permeation was considered. We assumed plug flow, isothermal operation, and constant permselectivity, and calculated the reaction equilibrium, the permeation of H_2_O, and the mass balance according to the membrane selectivity other than H_2_O. In addition, this simulation model does not consider the concentration polarization of the reaction tube in the radial direction.

## 3. Results and Discussion

**Figure 2:**
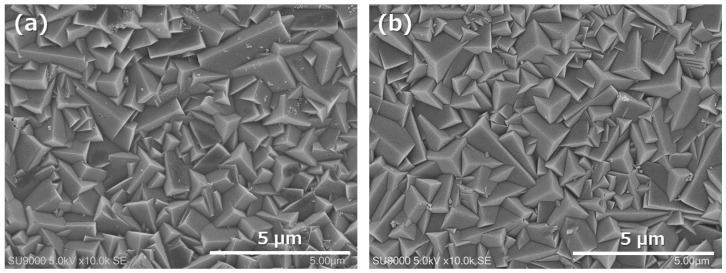
Surface SEM images of (**a**) the Si-rich LTA membrane and (**b**) conventional LTA membrane.

**Figure 3:**
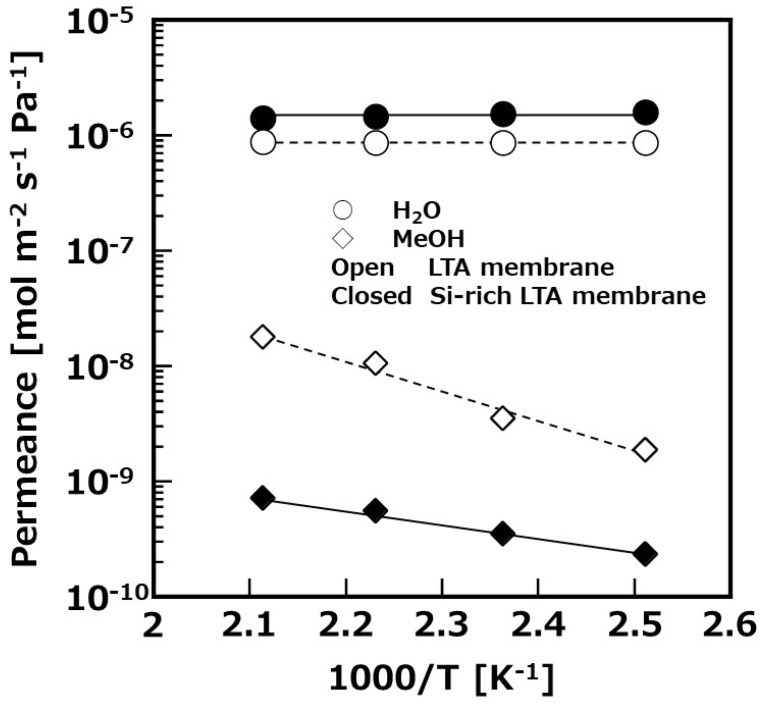
Temperature dependence of vapor permeation through synthesized Si-rich LTA membrane at 398–473 K in a 10/90 wt% vapor of H_2_O/MeOH.

The Si/Al ratio of the Si-rich LTA membrane was around 1.5 measured by SEM-EDX, and was similar to our previous work [25]. To compare conventional LTA membranes (Si/Al = 1), the framework of the membrane had a relatively high Si content. Surface SEM images are shown in Figure 2, comparing with conventional LTA membranes. The particle size and the morphology consisted of an Si-rich zeolitic layer that was approximately the same as that which consisted of LTA. The temperature dependence of H_2_O permselective performance through the synthesized Si-rich LTA and conventional LTA membranes is shown in Figure 3. H_2_O permeances of both membranes were approximately constant regardless of temperature. On the other hand, MeOH permeances were increased with increasing temperature. It is considered that the permeation mechanism of this membrane is based on adsorption separation owing to its hydrophilic nature. Thus, in a relatively high temperature range, the amount of H_2_O absorbed into pores of the membrane decreases, with the effect of inhibiting the permeation of MeOH, which becomes smaller. To compare between the permselective performance of the Si-rich LTA and LTA membranes, H_2_O permeance and H_2_O/MeOH selectivity of Si-rich LTA was higher than those of LTA membrane. The Si content in the Si-rich LTA membrane was increased compared with that in conventional LTA membrane from SEM-EDX measurement. The compensating cation (Na cation in this study) that inhibits H_2_O permeation through zeolitic pores is slightly less than the LTA membrane. Therefore, H_2_O permeance of the Si-rich LTA membranes was improved. In addition, H_2_O/MeOH selectivity of the Si-rich LTA membrane was also improved; thus, the hydrophilicity of the membrane was not significantly impaired. The Si-rich LTA membrane exhibited 1.5 × 10^−6^ mol m^−2^ s^−1^ Pa^−1^ of H_2_O permeance, and around 2000 of selectivity (H_2_O/MeOH) at 473 K, thus, this membrane was applied to the membrane reactor for methanol synthesis.

**Figure 4:**
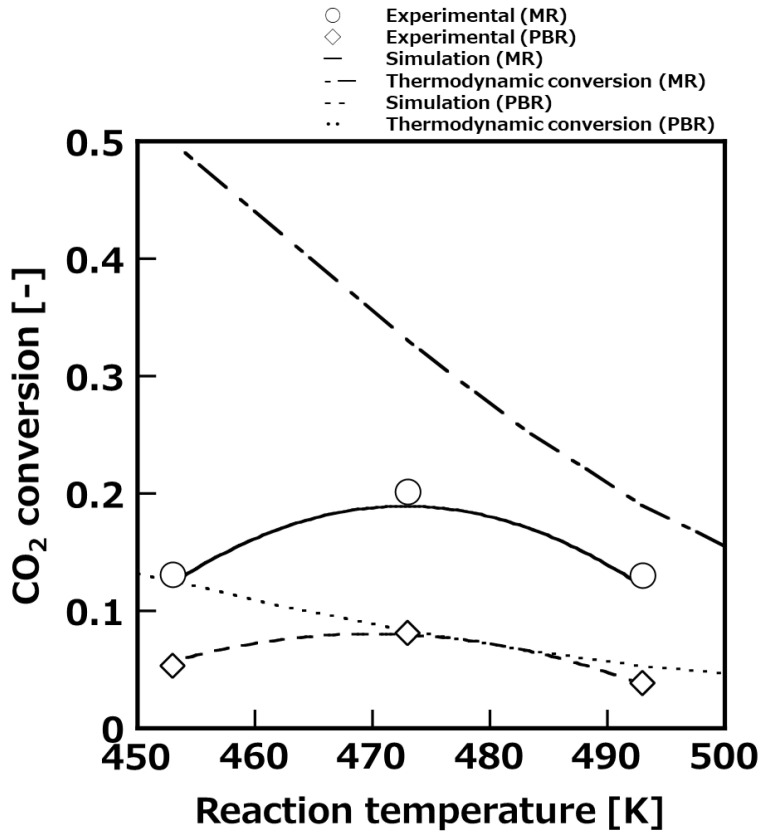
Temperature dependence of CO_2_ conversion to compare with membrane reactor (MR) and packed-bed reactor (PBR) at 1 MPa.

Figure 4 shows the temperature dependence of CO_2_ conversion at 1 MPa reaction pressure and the SV set at 200 h^−1^. For comparison, the results of the packed-bed reactor tests are also shown in this figure. In the results of the packed-bed reactor, the experimental and calculated conversion of CO_2_ were in good agreement in the temperature range 453–493 K, therefore, the constructed simulation model was appropriate. From this result, it was considered that the concentration polarization in the radial direction of the reactor was negligible. It was experimentally clarified that both the packed-bed reactor and membrane reactor showed the maximum CO_2_ conversion at a reaction temperature of 473 K. The CO_2_ conversion was 8% in the packed-bed reactor, while it was 20% in the membrane reactor. The thermodynamic equilibrium conversion of the packed-bed reactor and membrane reactor was calculated and compared with the experimental conversion to consider the reason that the CO_2_ conversion was shown maximum value at 473 K. The equilibrium conversion of the membrane reactor was calculated on the assumption that the water molecules were permeated to be the same partial pressure of water in feed and permeate side. When the reaction temperature was higher than 473 K, the CO_2_ conversion was decreased according to thermodynamic equilibrium conversion. On the other hand, the CO_2_ conversion was shown to have decreasing tendency because the catalyst activity was lower below 473 K. From these results, we successfully demonstrated that the CO_2_ conversion was improved owing to the removal of water molecules from the reaction system by the membrane reactor using the developed Si-rich LTA membrane. In addition, when the selectivity of H_2_O/raw material (CO_2_, CO, and H_2_) was 300, the experimental and calculated conversion of CO_2_ showed good agreement, therefore, the selectivity of the synthesized membrane was considered at least 300. The simulation results showed that the purity of MeOH after vapor–liquid separation was around 50% in the packed-bed reactor, however, the purity of MeOH was improved to around 95% in the membrane reactor that performed reaction and separation at the same time.

**Figure 5:**
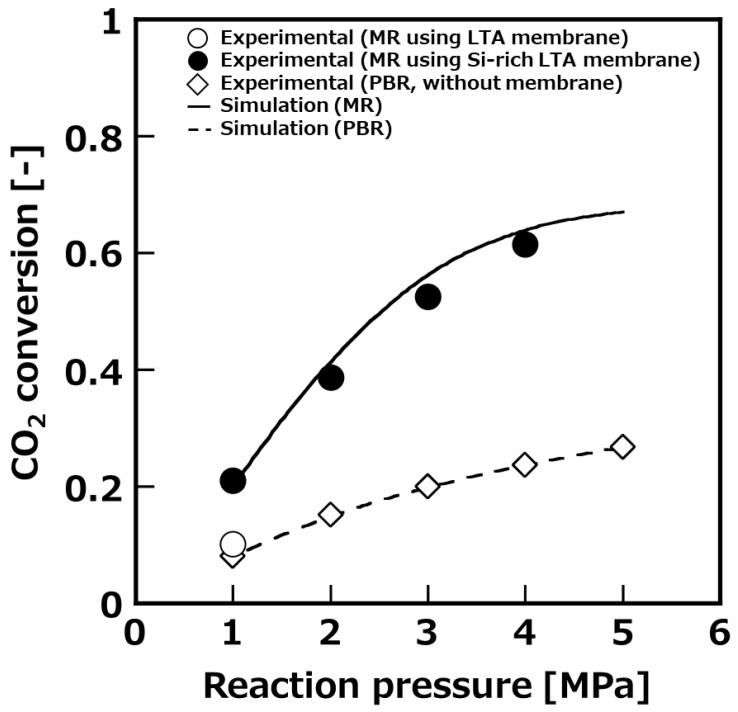
Pressure dependence of CO_2_ conversion to compare with membrane reactor (MR) and packed-bed reactor (PBR) (SV = 200 h^−1^).

Figure 5 shows the influence of reaction pressure on CO_2_ conversion at 473 K, which was the optimal reaction temperature resulted from Figure 4. The SV was set at 200 h^−1^. In both the packed-bed reactor and membrane reactor, the CO_2_ conversion improved as the reaction pressure increased. The membrane reactor using Si-rich LTA membrane showed a higher conversion in all pressure ranges compared with packed-bed reactor. In particular, at a reaction pressure of 4 MPa, the CO_2_ conversion of the packed-bed reactor was around 20%, while that of the membrane reactor was around 60%. On the other hand, a similar test was performed using LTA zeolite membrane, however, the CO_2_ conversion was approximately 10%, conversion that was approximately the same as with the packed-bed reactor. Water permselective performance of the LTA membrane was 8.7 x 10^−7^ mol m^−2^ s^−1^ Pa^−1^ of H_2_O permeance, and around 50 of H_2_O/MeOH selectivity at 473 K, which was lower than those of the Si-rich LTA membrane. In the membrane reactor test using the LTA membrane, we could not increase the reaction pressure above 1 MPa, because raw materials (H_2_ and CO_2_) were passing through the LTA membrane.

**Figure 6:**
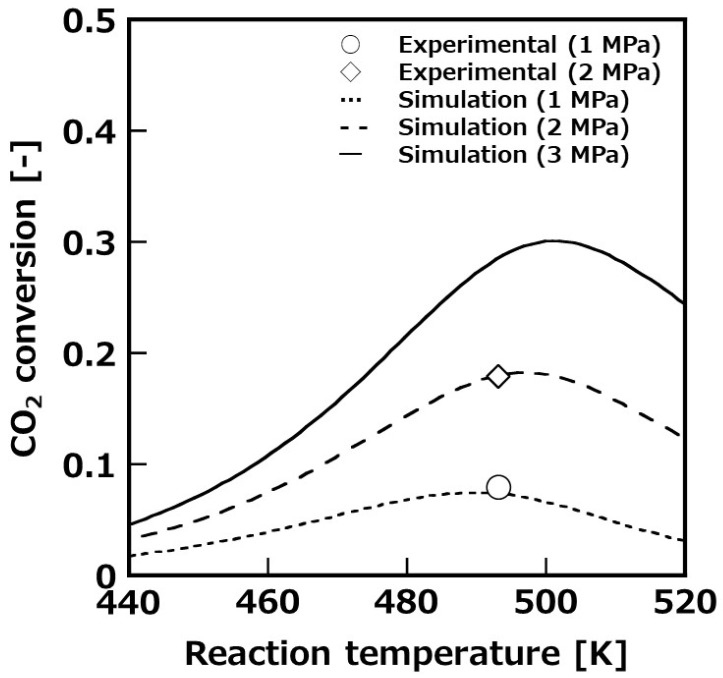
Experimental and simulation results of membrane reactor (SV = 1000 h^−1^).

Figure 6 shows the experimental and simulation results of the membrane reactor under a relatively high raw material feed rate (SV = 1000 h^−1^). In the simulation results, it was found that the CO_2_ conversion showed a maximum of around 493 K. The membrane reactor tests were conducted at 493 K in the reaction pressure range 1–2 MPa. When the reaction pressures were 1 and 2 MPa, the CO_2_ conversion showed 8 and 18%, respectively. In the case of the packed-bed reactor, those conversions were 5 and 8%, respectively. Therefore, the effect of the membrane reactor could be demonstrated even with a relatively high raw material feed rate.

## 4. Conclusions

We synthesized an Si-rich LTA zeolite membrane, and the membrane showed relatively high H_2_O permselective performance compared with a conventional LTA membrane. The methanol synthesis membrane reactor test using synthesized an Si-rich LTA membrane was conducted under the reaction conditions with SV = 200 and 1000 h^−1^ and reaction temperature of 453–493 K. Higher CO_2_ conversion of the membrane reactor was obtained than that of conventional packed-bed reactor because H_2_O molecules were extracted from the reaction system. In particular, the CO_2_ conversion of membrane reactor achieved around 60% at 4 MPa, which was three times higher than that of the packed-bed reactor under the reaction conditions of SV = 200 h^−1^, and reaction temperature of 473 K. The constructed simulation model for membrane reactor was able to reproduce the experimental results in this study. For practical use, it is necessary to increase the effective membrane area of the Si-rich LTA membrane, and develop an optimal structure of membrane reactor module for methanol synthesis.

## Data Availability

Not applicable.
